# Mapping the Human Herpesvirus 6B Transcriptome

**DOI:** 10.1128/JVI.01335-20

**Published:** 2021-04-26

**Authors:** Annie Gravel, Wes Sanders, Éric Fournier, Arnaud Droit, Nathaniel Moorman, Louis Flamand

**Affiliations:** a Division of Infectious Disease and Immunity, CHU de Québec Research Center, Quebec City, Québec, Canada; b Department of Microbiology and Immunology, UNC School of Medicine, Chapel Hill, North Carolina, USA; c Division of Endocrinology and Nephrology, CHU de Québec Research Center, Quebec City, Québec, Canada; d Department of Microbiology, Infectious Disease and Immunology, Faculty of Medicine, Université Laval, Quebec City, Québec, Canada; University of Arizona

**Keywords:** HHV-6B, RNA-seq, herpesviruses, transcriptomic

## Abstract

The “omics” revolution of recent years has simplified the study of RNA transcripts produced during viral infection and under specific defined conditions. In the quest to find new and differentially expressed transcripts during the course of human herpesvirus 6B (HHV-6B) infection, we made use of large-scale RNA sequencing to analyze the HHV-6B transcriptome during productive infection of human Molt-3 T cells. Analyses were performed at different time points following infection, and specific inhibitors were used to classify the kinetic class of each open reading frame (ORF) reported in the annotated genome of the HHV-6B Z29 strain. The initial search focused on HHV-6B-specific reads matching new HHV-6B transcripts. Differential expression of new HHV-6B transcripts was observed in all samples analyzed. The presence of many of these new HHV-6B transcripts was confirmed by reverse transcriptase PCR and Sanger sequencing. Many of these transcripts represented new splice variants of previously reported open reading frames (ORFs), including some transcripts that have yet to be defined. Overall, our work demonstrates the diversity and the complexity of the HHV-6B transcriptome.

**IMPORTANCE** RNA sequencing (RNA-seq) is an important tool for studying RNA transcripts, particularly during active viral infection. We made use of RNA-seq to study human herpesvirus 6B (HHV-6B) infection. Using six different time points, we were able to identify the presence of differentially spliced genes at 6, 9, 12, 24, 48, and 72 h postinfection. Determination of the RNA profiles in the presence of cycloheximide (CHX) or phosphonoacetic acid (PAA) also permitted identification of the kinetic class of each ORF described in the annotated GenBank file. We also identified new spliced transcripts for certain genes and evaluated their relative expression over time. These data and next-generation sequencing (NGS) of the viral DNA have led us to propose a new version of the HHV-6B Z29 GenBank annotated file, without changing ORF names, to facilitate trace-back and correlate our work with previous studies on HHV-6B.

## Abstract (precis)

RNA sequencing (RNA-seq) is an important tool for studying RNA transcripts, particularly during active viral infection. We made use of RNA-seq to study human herpesvirus 6B (HHV-6B) infection.

## INTRODUCTION

Human herpesvirus 6B (HHV-6) is a member of the genus *Roseolovirus*, within the *Betaherpesvirinae* subfamily of the *Herpesviridae* family. HHV-6B is acquired by greater than 90% of the population by the age of three and is the etiologic agent of Roseola, also called sixth disease or *exanthema subitum* (1). Roseola is usually resolved without any treatment and/or consequences, but febrile seizures have been observed in more than 10% of acute infections (2). HHV-6B reactivation is also frequent in hematopoietic stem cell transplant recipients, with serious medical consequences, including encephalitis and acute graft versus host disease (3). As with all herpesviruses, HHV-6B establishes latency following primary infection. During latency, most herpesviruses maintain their genome as episomes. The presence of HHV-6B viral episomes during latency awaits experimental confirmation. However, integration of HHV-6A/B into human chromosomes can be found in approximately 1% of the world population (4–6). HHV-6A/B can integrate in the telomeres of several distinct chromosomes (5, 7, 8). Chromosomally integrated HHV-6A/B can be inherited, resulting in individuals having at least one copy of the viral genome in every cell of their body. Viral integration into human telomeres has been suggested as an alternative form of latency. The repercussions of chromosomally integrated HHV-6A/B on an individual’s health are unknown, but we have recently demonstrated that these subjects are more likely to develop angina pectoris (9). Recent studies have also shown that chromosomally integrated HHV-6A/B may spontaneously express certain viral genes (U90 and U100), and iciHHV-6A/B^+^ individuals have greater antibody responses against these viral genes than matched controls (10).

The HHV-6A/B genome comprises a 160-kb linear doubled-stranded DNA molecule (11, 12). A unique segment (U) is flanked by direct repeats (DR) of approximately 8 kb. The nucleotide sequences and annotations of HHV-6A/B genomes were obtained in the 1990s. The nucleotide sequence of the HHV-6A U1102 strain was the first published in 1995 (12). In this Ugandan strain, a total of 119 open reading frames (ORFs) were described, corresponding to 102 distinct genes. The nucleotide sequence was obtained following the sequencing of a library of viral genome segments cloned in plasmids, cosmids, and bacteriophages. Four years later, the nucleotide sequences of HHV-6B Z29 and HHV-6B HST, from Zaire and Japan, respectively, were published (11, 13). The nucleotide sequence of the HHV-6B Z29 strain predicts 119 unique ORFs comprising 97 unique genes, whereas the nucleotide sequence of the HHV-6B HST strain indicates 115 potential ORFs. These 2 sequences were determined by plasmid clones, overlapping PCR fragments, and sequencing. In 2013, Gravel et al. published the nucleotide sequence of a low-passage-number strain, HHV-6A GS (14). This strain was the first isolated HHV-6A in 1986 and originated from the United States. The nucleotide sequence was determined by Illumina sequencing, and gaps were filled by PCR. This sequence carries 88 putative genes. All annotated sequences were based on ORF analysis using conventional translation start and stop codons.

Innovative “omics” approaches in recent years have made high-throughput sequencing more available and less expensive, making large genome sequencing and RNA sequencing (RNA-seq) possible. In the last 2 years, a significant amount of data has become available, with the determination of numerous genomes, transcriptomes, translatomes, and proteomes for many species and pathogens. Herpesviruses have proven no exception, with many omics data sets available for cytomegalovirus, gammaherpesviruses, herpes simplex virus (HSV), and even HHV-6A/B. In recent months, a group from Israel reported novel and conserved genomic features for HHV-6A/B using RNA-seq and ribosome profiling (Ribo-seq) (15). Using these two techniques, they were able to accurately determine the translation initiation sites of previously annotated genes and to identify hundreds of new ORFs. Novel splice junctions were mapped, and novel highly abundant viral long noncoding RNAs were identified. They also proposed systematic annotations of the two viruses following their previous annotation of the human cytomegalovirus (hCMV) (16).

The objective of this study was to determine the RNA profile during the course of an active HHV-6B Z29 infection in the Molt-3 T-cell line. We also wanted to determine the RNA profile of HHV-6B Z29 infection in the presence of cycloheximide (CHX) and phosphonoacetic acid (PPA), two inhibitors that permit the identification of the kinetic class of each gene. Finally, we defined new spliced variants of several genes, identified new transcripts, and proposed a new annotation for HHV-6B Z29 that is built on the current and widely used reference sequence.

## RESULTS

### Coverage and splicing patterns.

**FIG 1:**
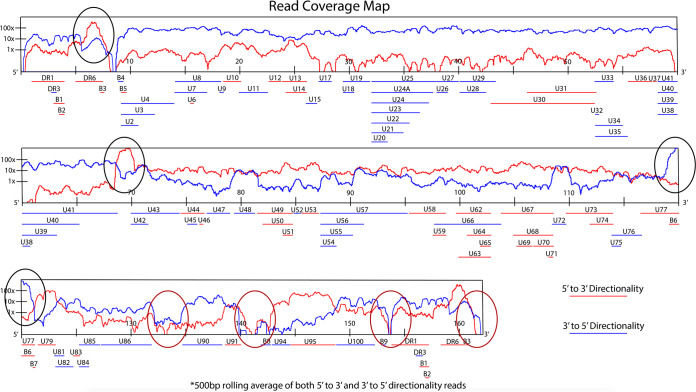
HHV-6B genome read coverage map. A map showing coverage obtained in the 5′ to 3′ direction (red line) and the 3′ to 5′ direction (blue line) of the HHV-6B genome. Black circles show regions where higher coverage was obtained in either direction. Red circles show regions where lower coverage was obtained in either direction.

To explore the transcriptome of Molt-3 cells infected with HHV-6B, we performed an infection time course study with samples taken at 6, 9, 12, 24, 48, and 72 h postinfection. RNA was extracted and analyzed by RNA-seq. The 72-h postinfection sample was initially used to analyze the read coverage obtained using our RNA-seq protocol. As observed in Fig. 1, a coverage of 100× was obtained for most of the genome, indicating efficient transcription throughout the genome. Some regions showed very high coverage, e.g., the DR6 region, the forward direction of the U41-U42 region, and the reverse orientation of the U77 gene. In contrast, genomic repeat regions, exemplified by telomeric repeats, R2 and R3, showed less than 1× or no coverage, indicating that these regions are not efficiently transcribed.

### Relative expression and transcript kinetic class over time.

**FIG 2:**
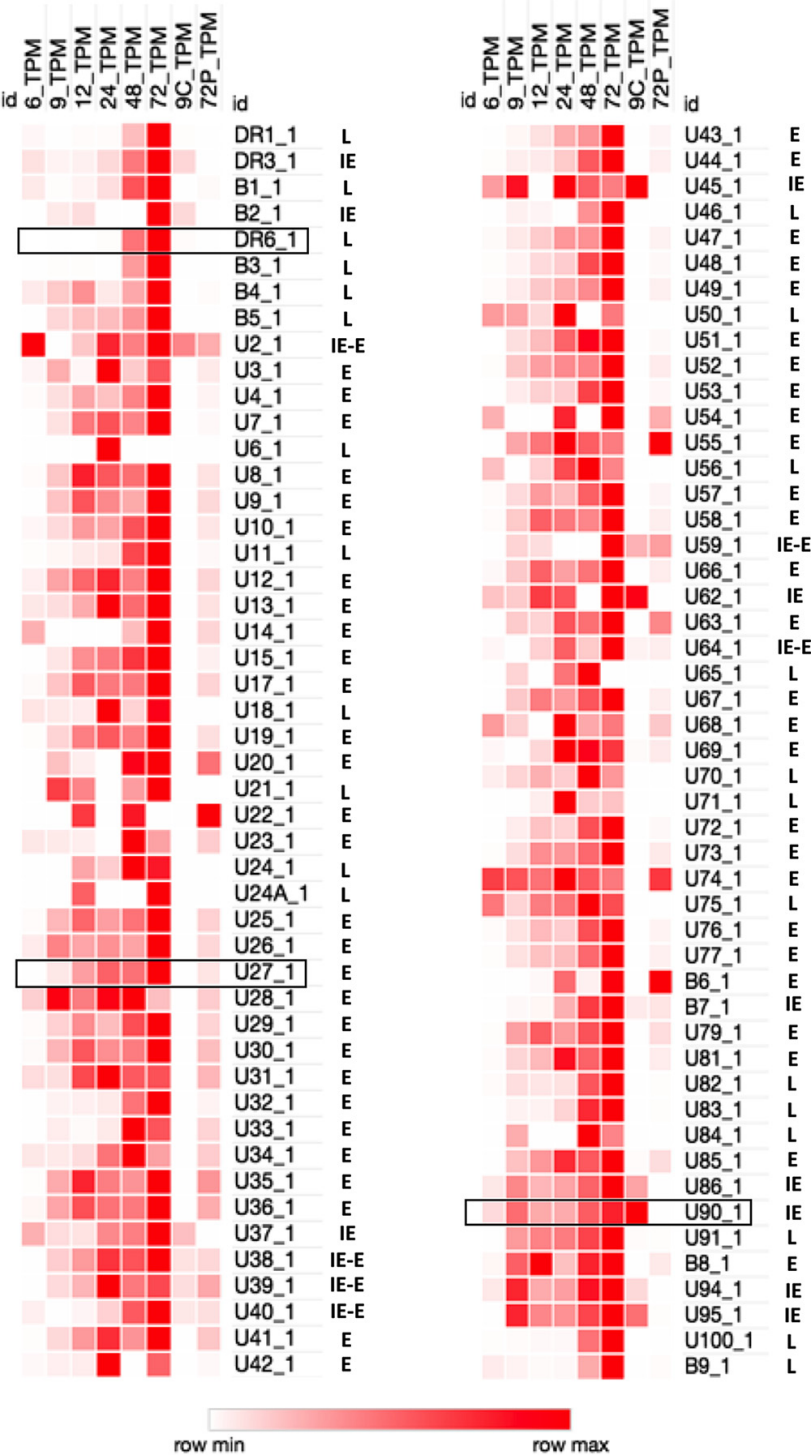
Detailed heat map of HHV-6B transcripts. Relative abundance of each of the HHV-6B transcripts described in the reference genome annotation AF157706 is shown. Each column represents a given condition (6, 9, 12, 24, 48, 72 h postinfection; 9C, which represents 9 h postinfection in the presence of CHX; and 72P, which represents 72-h postinfection in the presence of PAA), and each row represents a described transcript. Black boxes exemplify the 3 classes of genes: immediate-early genes represented by U90, early genes represented by U27, and late genes represented by DR6.

Using data from across the time course of infection, we generated a heat map showing the relative expression of each ORF described in the annotated HHV-6B genome (NCBI accession number AF157706). As shown in Fig. 2, we found that particular genes, such as U90 (IE1), were expressed in abundance throughout the course of infection. For other genes, such as U27, expression began at 9 h postinfection. Genes such as DR6 were only expressed from 48 h postinfection. To complete our analysis, we performed RNA-seq on RNA extracted from HHV-6B-infected Molt-3 cells in the presence of CHX, an inhibitor of protein synthesis (9C in Fig. 2). As shown in Fig. 2, the U90 (IE1) gene was highly expressed in the presence of CHX and can be classified as belonging to the immediate-early (IE) kinetic class. The DR3, B2, U2, U37, U38, U39, U40, U45, U59, U62, U64, B7, U86 (IE2), U94, and U95 genes were also expressed in the presence of CHX, categorizing these genes in the IE kinetic class. A similar experiment was performed in the presence of phosphonoacetic acid (PAA), an inhibitor of viral DNA polymerase (72P in Fig. 2). We characterized early (E) genes as those expressed in the presence of PAA but not in the presence of CHX. The U20, U22, U54, U55, U63, U74, and B6 genes were the most highly expressed genes in this kinetic class. Genes that were not expressed in the presence of CHX or PAA were categorized as belonging to the late (L) kinetic class. Examples include the DR1, DR6, U11, U18, and U100 genes.

**FIG 3:**
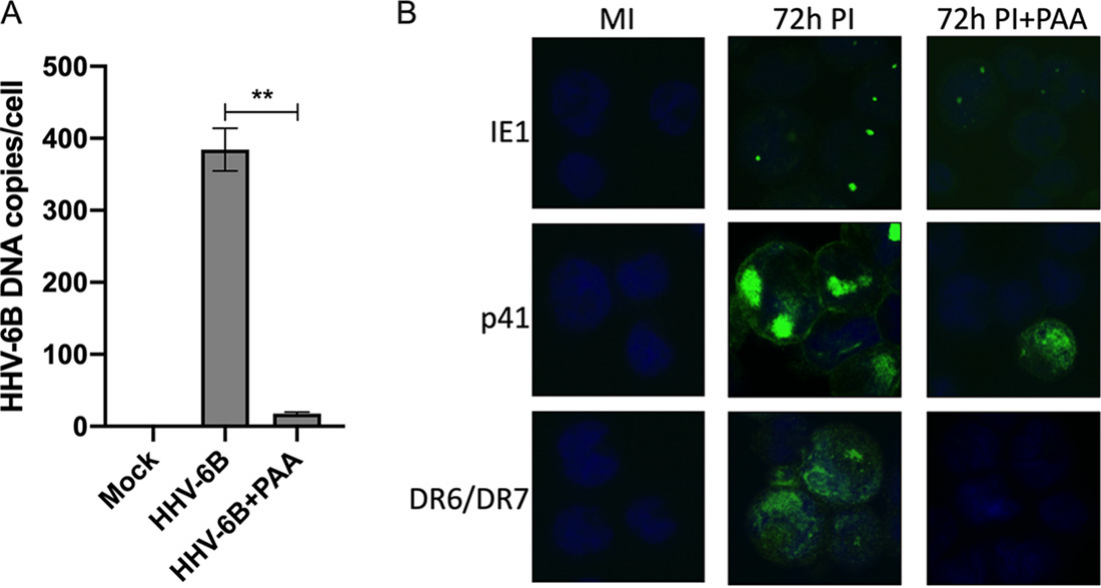
Effect of PAA on HHV-6B copies per cell and protein expression. (A) Molt-3 cells were infected for 4 h and 72 h with HHV-6B in the absence or presence of 100 μg/ml PAA. Mock-infected (MI) Molt-3 cells were used as controls. Genomic DNA was isolated and used in a ddPCR experiment using an U67-U68/hRPP30 assay to determine the viral copy number per cell. Data presented are the means from three independent experiments and were analyzed using unpaired *t* test with Welch’s correction (**, *P* < 0.001). (B) Molt-3 cells were infected for 72 h with HHV-6B in the absence or presence of 100 μg/ml PAA. Mock-infected (MI) Molt-3 cells were used as controls. Cells were fixed and labeled with Alexa-488-labeled anti-IE1, anti-p41, and anti-DR6/7 antibodies, followed by Alexa-488-labeled anti-mouse IgG. Slides were then examined under a fluorescence microscope.

The efficiency of PAA treatment was determined by quantifying viral DNA copies at 72 h postinfection in the absence or in the presence of PAA. As shown in Fig. 3A, a 95% reduction in viral DNA copy number per cell was observed in infected cells treated with PAA relative to infected and untreated cultures. Protein expression of one gene per kinetic class was evaluated in the presence of PAA. An immunofluorescence assay was used to detect the IE1 (U90), p41 (U27), and DR6 proteins. As shown in Fig. 3B, we detected the IE1 (U90) and p41 (U27) proteins in HHV-6B-Molt-3-infected cells 72 h postinfection in both the presence and absence of PAA. However, the DR6 protein was only detectable in the absence of PAA, confirming that DR6 is a late gene.

### Sequence analysis and comparison with the published sequence.

Next, we analyzed NGS data obtained from HHV-6B Z29 viral DNA and RNA-seq data obtained from HHV-6B-Molt-3 cells infected for 72 h. The sequence was first analyzed to identify nucleotide mismatches from the nucleotide sequence under NCBI accession number AF157706; 95 mismatches in the nucleotide sequence were identified (Table 1). Thirty-three mismatches (highlighted in orange) were previously found in the MF994829.1 sequence and reported by Finkel et al., while 12 additional mismatches (highlighted in pink) were restricted to the MF994829.1 sequence (17) (Table 1). Of these 95 mismatches, 26 were located in noncoding regions of genes annotated in the reference sequence, leaving 69 mismatches located in the coding regions affecting 74 amino acids of coding sequence (Table 1). Twenty of these 74 differences did not affect the amino acid coding sequence of the corresponding ORF (yellow), 16 differences were conservative mutations (green), 37 differences were nonconservative mutations, and one amino acid difference changed a tryptophan into a stop codon (blue) (Table 1). Six insertions were found in noncoding sequences, while two were found in the B9 coding sequence (Table 2). These insertions are present in the vast majority of HHV-6B sequences found in GenBank. They were previously identified in MF994829.1 (17), and five were referenced by Finkel et al. (15). One deletion was identified in the intergenic region between B6 and B7 (Table 2). This deletion removed a T from the original sequence at bp 119973. Comparison of all HHV-6B sequences found in GenBank supports that this is a genuine deletion. A number of reads cover this region, and most of the sequences found in GenBank have T deleted at this location. This deletion was also found in the MF994829.1 sequence and mentioned in the study by Finkel et al. (15, 17).

**TABLE 1:**
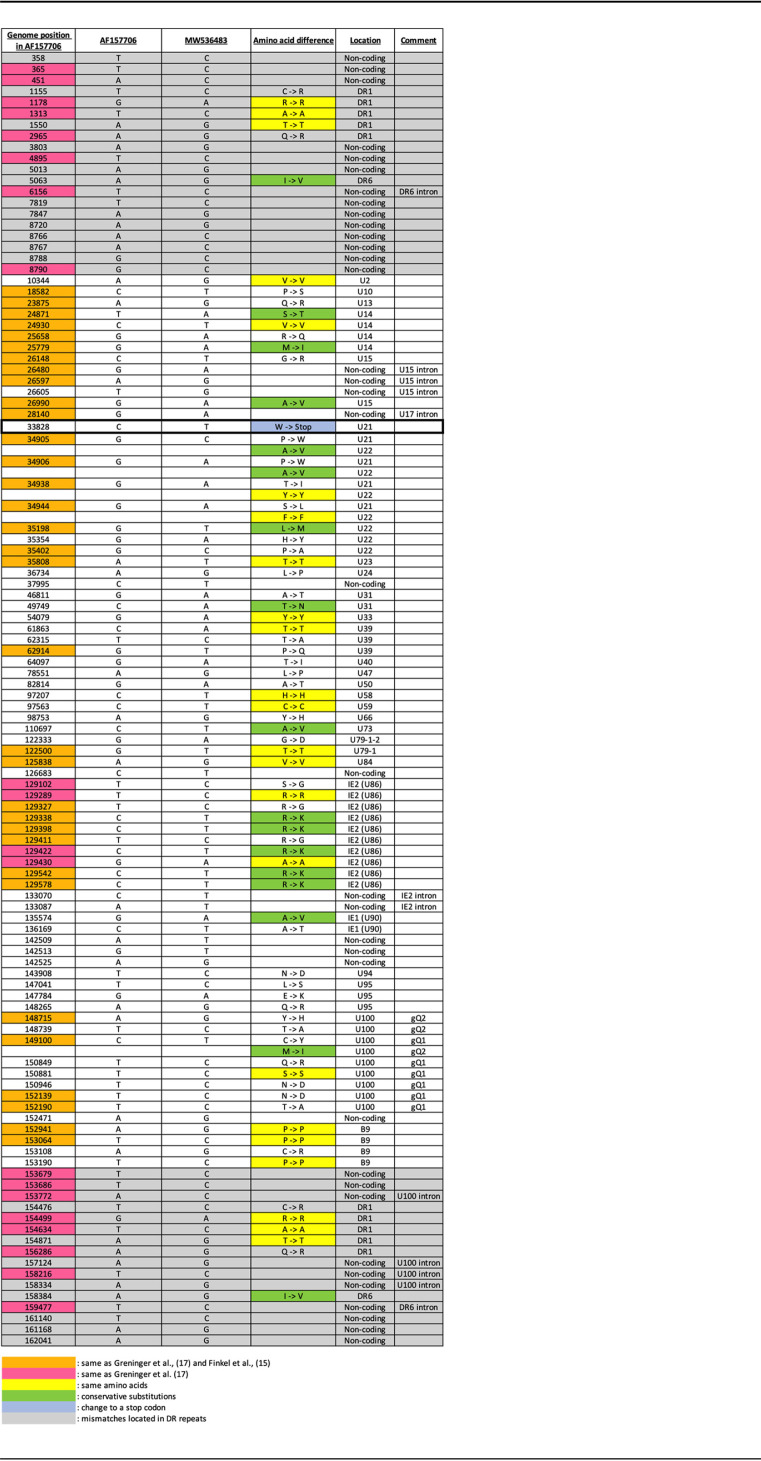
Nucleotide mismatches detected in the HHV-6B sequence

**TABLE 2:**
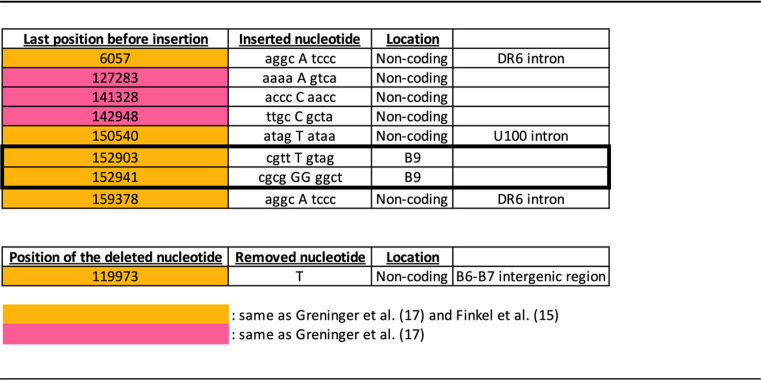
Insertions and deletions found in the HHV-6B sequence

**FIG 4:**
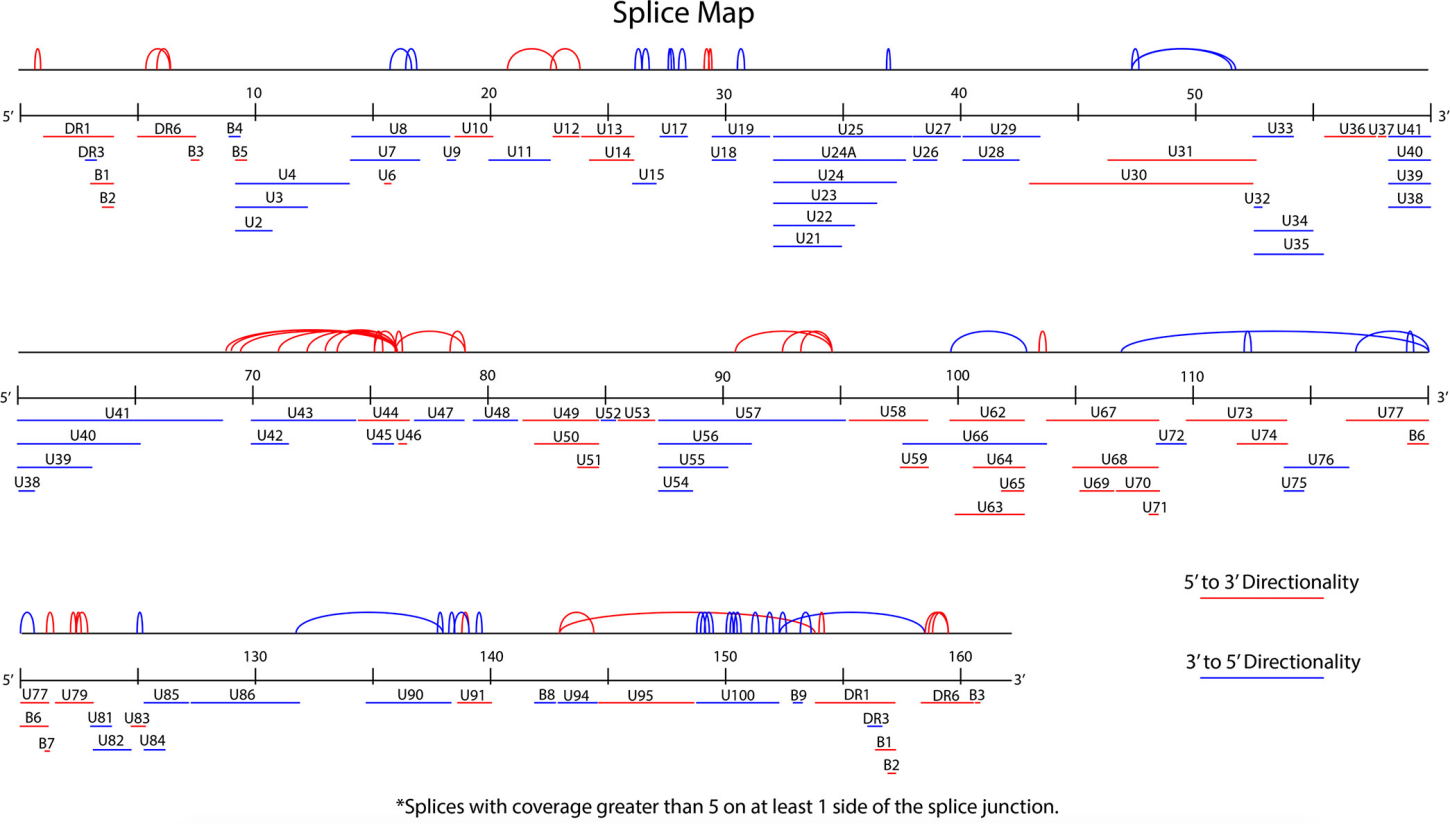
HHV-6B splice map. The map shows the different splicing found in the RNA-seq analysis at 72 h postinfection. The red lines show 5′ to 3′ directionality, and the blue lines represent 3′ to 5′ directionality.

**FIG 5:**
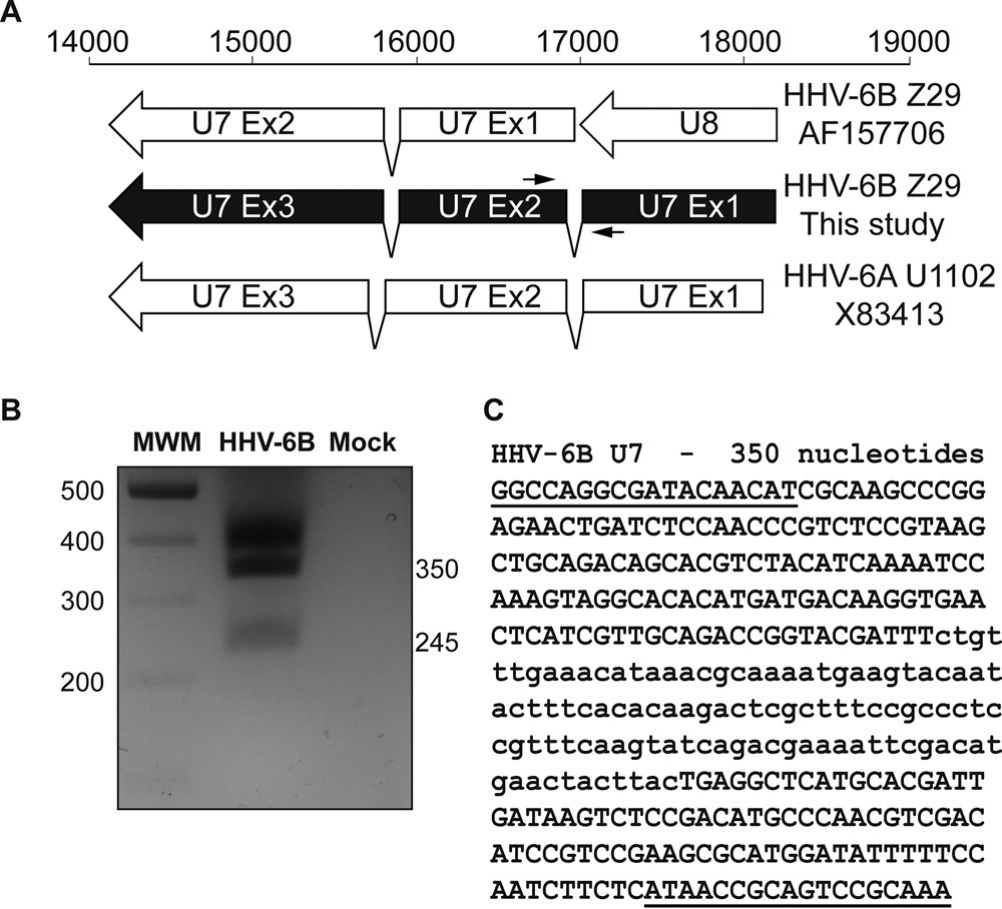
U7-U8 gene region of HHV-6B. (A) Read analysis revealed a new arrangement for the U7-U8 gene region of HHV-6B, which shows similarity to the U7 arrangement of HHV-6A U1102 (reference genome annotation X83413). The AF157706-Z29 drawing represents the description found in annotated genome AF157706 for HHV-6B Z29. (B) PCR primers were designed to surround the new splicing region (black arrows in panel A). PCR was performed as described in Materials and Methods. PCR products were analyzed on a 2% agarose gel. (C) PCR products were extracted from the gel, purified, and sequenced. Primer sequences are underlined, and the intron sequence is presented in lowercase.

Our attention then turned to splice variants of the virus transcriptome. We used our RNA-seq data to generate a splice map in relation to the annotated genome (Fig. 4). A number of splicing events occurred around the U44 gene: long-range splicing events in the reverse orientation of the U77 gene, in the same orientation as the U95 gene, and the previously described multispliced transcripts of U86, U90, and U100 (Fig. 4). We first concentrated on the U7-U8 genes. U7 and U8 are described as two genes in the Z29 strain of HHV-6B (NCBI accession number AF157706) (Fig. 5A). Our RNA-seq data revealed that U8 is part of exon 1 of the U7 gene, since reads were found that spanned the exon-intron-exon region and consensus splicing acceptor and donor sites. The gene arrangement described here is similar to that of the HHV-6A U1102 U7 gene (NCBI accession number X83413) (Fig. 5A). To confirm these data, we designed PCR primers spanning the new intronic region and performed reverse transcriptase PCR (RT-PCR) on a new RNA sample from HHV-6B-Molt-3-infected cells (Fig. 5B). As shown in Fig. 5B, we observed a 350-bp PCR product corresponding to the unspliced version of the cDNA and a PCR product of 245 bp corresponding to the spliced version of the U7 gene. The two PCR products were isolated from the agarose gel, purified, and sequenced to confirm our findings. The DNA sequence is presented in Fig. 5C, showing the intronic region in lowercase. This new arrangement for U7 and U8 ORFs was described by Finkel et al. (15).

**FIG 6:**
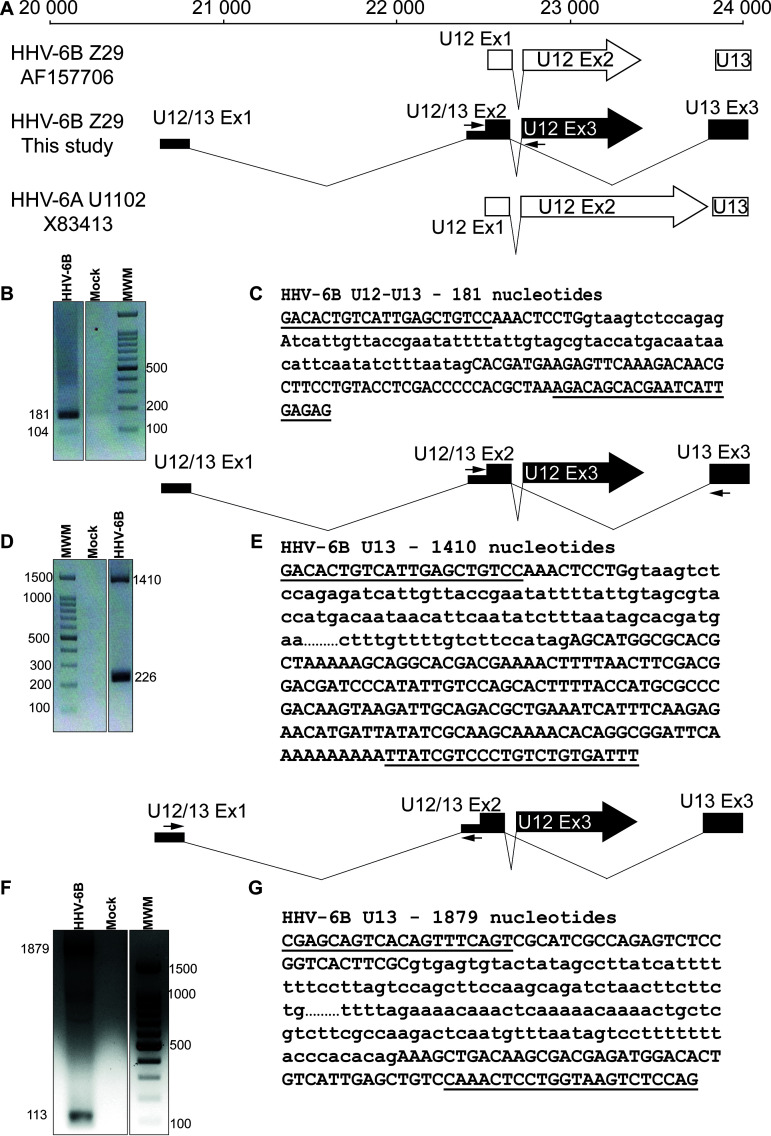
U12-U13 region of HHV-6B. (A) Read analysis revealed a new arrangement for the U12 and U13 genes of HHV-6B. The AF157706-Z29 drawing represents the description found in annotated genome AF157706 for HHV-6B Z29. (B) PCR primers were designed to surround the splice region between the noncoding and coding exons (black arrows in panel A). PCR was performed as described in Materials and Methods. PCR products were analyzed on a 2% agarose gel. (C) PCR products were extracted from the gel, purified, and sequenced. Primer sequences are underlined, and the intron sequence is presented in lowercase. (D) PCR primers were designed to surround the splice region between the coding exons of the U13 gene (black arrows). PCR was performed as described in Materials and Methods. PCR products were analyzed on a 2% agarose gel. (E) PCR products were extracted from the gel, purified, and sequenced. Primer sequences are underlined, and the intron sequence is presented in lowercase. The intron sequence was cut (…) to fit the figure. (F) PCR primers were designed to surround the splice region between U12-U13 exon 1 and exon 2 (black arrows). PCR was performed as described in Materials and Methods. PCR products were analyzed on a 2% agarose gel. (G) PCR products were extracted from the gel, purified, and sequenced. Primer sequences are underlined, and the intron sequence is presented in lowercase. The intron sequence was cut (…) to fit the figure.

The U12-U13 region was the next region to be investigated. In the NCBI entry with accession number AF157706, the U12 gene is described as two exons separated by an intron (Fig. 6A). In the HHV-6A U1102 genome annotation, the U12 gene is also described as two exons separated by an intron, but the second exon is longer than that described for HHV-6B (Fig. 6A). HHV-6B Z29 is exceptional in this instance, as most other HHV-6B strains sequenced to date do not have this stop codon and resemble the HHV-6A version of U12 (18). The 72-h postinfection RNA-seq data revealed a limited number of reads that covered the intron present between the two exons described for U12 (Fig. 6A). We confirmed these data using RT-PCR and sequencing, but the 104-bp PCR product was very faint, indicating very low abundance of this transcript (Fig. 6B and C). RNA-seq data also indicated that the first exon of U12 is part of the U13 gene (Fig. 6E). Splicing between exons 2 and 3 of U13 (Fig. 6D and E) and the splicing event in the noncoding region of U12-U13 (Fig. 6F and G) were confirmed by RT-PCR and sequencing. Therefore, U12 and U13 share both the noncoding exon 1 and exon 2 but have distinct third exons. This observation was partially described by Finkel et al. (15).

**FIG 7:**
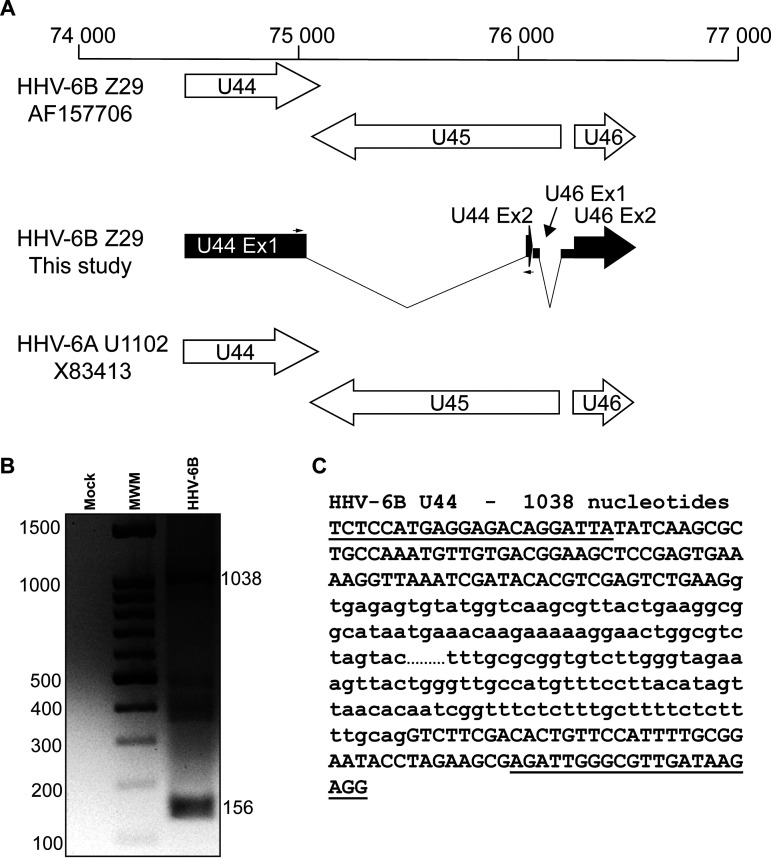
U44 gene region of HHV-6B. (A) Read analysis revealed a new arrangement for the U44 gene region of HHV-6B that is not described in HHV-6A U1102 (reference genome annotation X83413). The AF157706-Z29 drawing represents the description found in annotated genome AF157706 for HHV-6B Z29. (B) PCR primers were designed to surround the new splicing region (black arrows in panel A). PCR was performed as described in Materials and Methods, and PCR products were analyzed on a 2% agarose gel. (C) PCR products were extracted from the gel, purified, and sequenced. Primer sequences are underlined, and the intron sequence is presented in lowercase. The intron sequence was cut (…) to fit the figure.

The RNA-seq data obtained for the region spanning U44 to U46 were of particular interest, since many reads and splicing events were observed. The HHV-6B Z29 annotation described the transcription of the U44 and U46 genes in the 5′ to 3′ direction, whereas the U45 gene was transcribed in the reverse direction (Fig. 7A). No intron was described for these three genes in either HHV-6B or HHV-6A (Fig. 7A). The presence of a long transcript spanning U44 and portions of U45 and U46 was revealed by RNA-seq data (Fig. 7A). The presence of this new long intron in the U44 gene was confirmed by RT-PCR and sequencing of PCR products (Fig. 7B and C).

**FIG 8:**
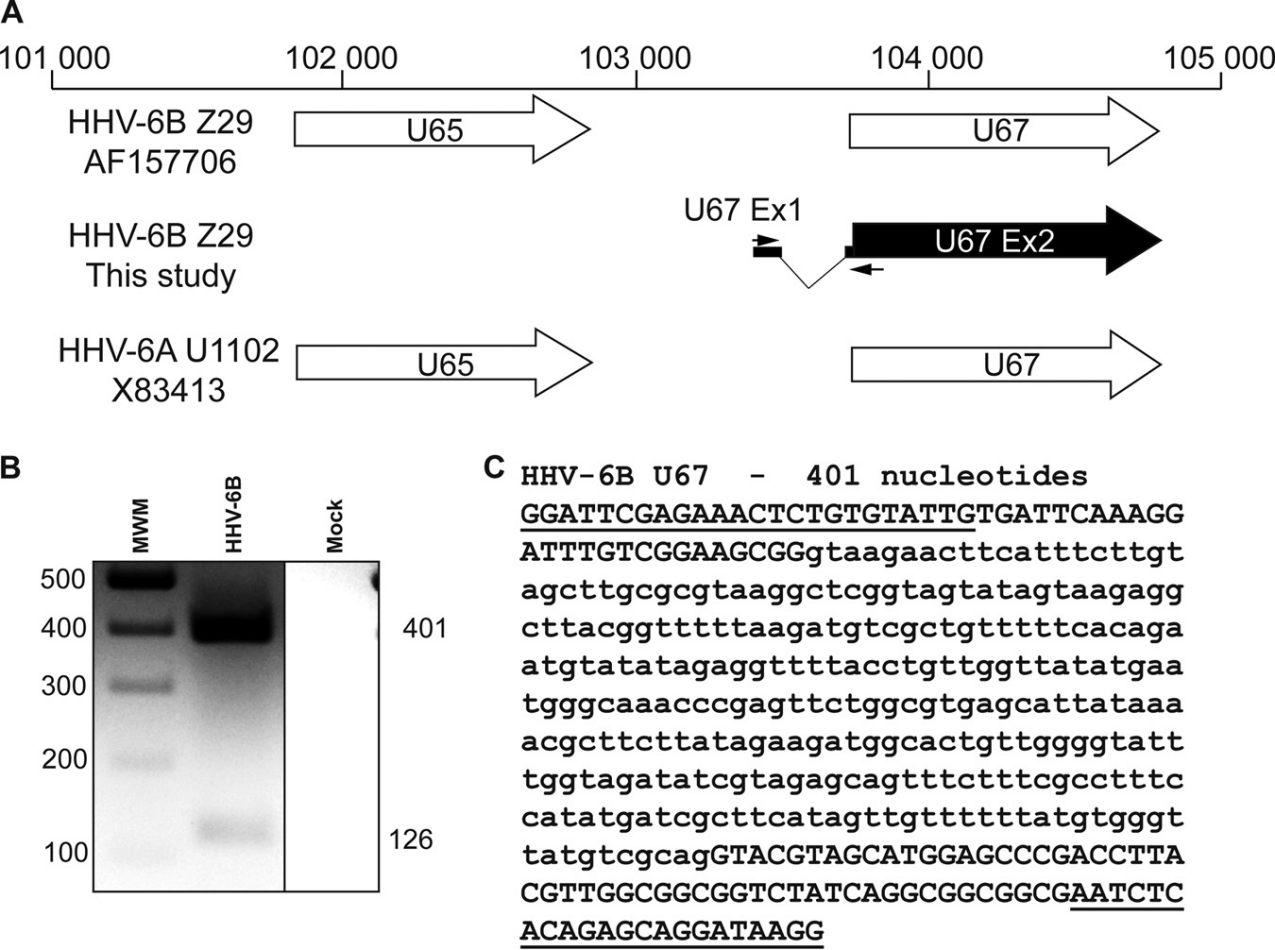
U67 gene region of HHV-6B. (A) Read analysis revealed a new arrangement for the U67 gene region of HHV-6B that is not described in HHV-6A U1102 (reference genome annotation X83413). The AF157706-Z29 drawing represents the description found in annotated genome AF157706 for HHV-6B Z29. (B) PCR primers were designed to surround the new splicing region (black arrows in panel A). PCR was performed as described in Materials and Methods, and PCR products were analyzed on a 2% agarose gel. (C) PCR products were extracted from the gel, purified, and sequenced. Primer sequences are underlined, and the intron sequence is presented in lowercase.

The U65-U67 region of HHV-6B was also examined. In the NCBI entry with accession number AF157706, the U67 gene is annotated as a single exon, with no intron (Fig. 8A). The analysis of our RNA-seq data revealed a spliced variant of this gene with a new noncoding exon in the 5′ region of the described U67 (Fig. 8A). These results were confirmed by RT-PCR and sequencing of the PCR products (Fig. 8B and C). This new spliced U67 gene was not described in the annotated HHV-6A U1102 genome.

### Other spliced transcripts.

**FIG 9:**
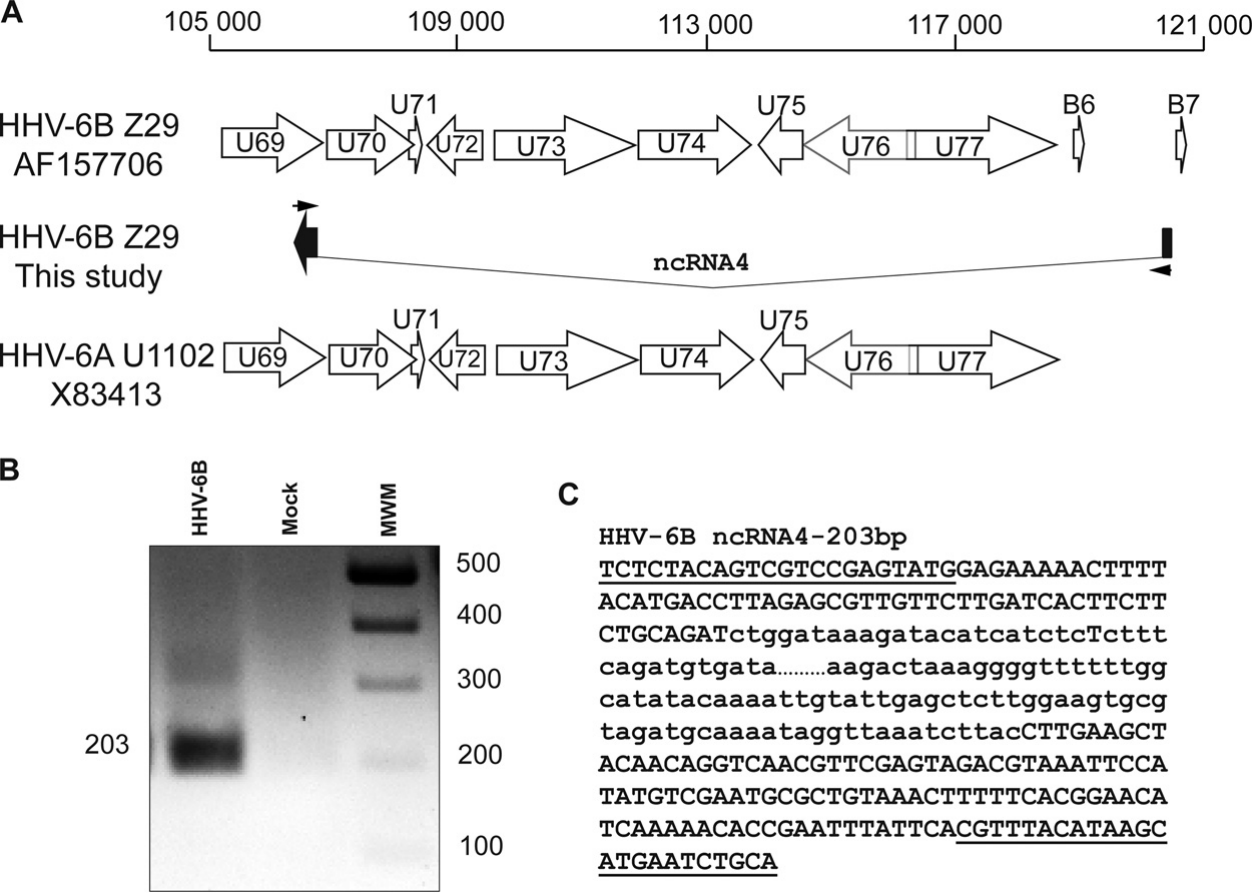
ncRNA4 of HHV-6B. (A) Read analysis revealed an undescribed ncRNA4 in the region spanning the U69 to B7 genes. The AF157706-Z29 drawing represents the description of annotated genome AF157706 for HHV-6B Z29. (B) PCR primers were designed to surround the new splicing region (black arrows in panel A). PCR was performed as described in Materials and Methods, and PCR products were analyzed on a 2% agarose gel. (C) PCR products were extracted from the gel, purified, and sequenced. Primer sequences are underlined, and the intron sequence is presented in lowercase. The intron sequence was cut (…) to fit the figure.

The region spanning U69 to B7 was investigated next. Our RNA-seq data revealed an intron that spanned more than 13,000 bp (Fig. 9A). This intron is part of a transcript that originated between the B6 and B7 genes and ended in the reverse orientation of the U69 gene. The presence of this intron was confirmed by RT-PCR and sequencing of the PCR products (Fig. 9B and C). Short exons flanked this intron, giving an ORF of 87 bp. While this transcript is of limited abundance, it was present in the 48- and 72-h postinfection samples. It is currently unknown whether this spliced transcript translates into a functional protein or whether it is one of the noncoding RNAs (sncRNAs or lncRNAs) found in many herpesvirus genomes. In 2020, Finkel et al. reported the presence of at least 3 lncRNA in the HHV-6B genome (15). We confirmed the presence of lncRNA3 in our data and that this transcript was expressed as early as 9 h postinfection. This transcript was continuously expressed from 9 h up to 72 h postinfection and was expressed in the presence of PAA, suggesting that it was an early transcript whose expression did not require viral DNA replication.

## DISCUSSION

The omics revolution of recent years has brought tremendous possibilities to the study of large genomes of any origin at different levels. Here, we determined the RNA profile of an HHV-6B Z29 active infection at 6, 9, 12, 24, 48, and 72 h postinfection and at 9 h and 72 h postinfection in the presence of CHX and PAA, respectively.

Using data from the 72-h postinfection RNA-seq, we constructed a splice map for the HHV-6B genome (Fig. 4). A number of splicing events were observed in the 68,000- to 78,000-bp region of the genome, and all spliced events were oriented in the forward direction (Fig. 4). This observation coincided with the very high coverage obtained in the forward direction of the 69,000-bp region of the genome (Fig. 1) and could be explained by the fact that this location is the origin of replication (OriLyt) of HHV-6B. It was previously demonstrated that sequences deriving from around the OriLyt of rat and mouse cytomegalovirus, and herpesvirus simplex 1 (HSV-1) and HSV-2, can act as RNA primers for replication of the viral genome (19–21). Published studies report that miRNAs are in the OriLyt region. In 2011, Tuddenham et al. identified miRNAs and other small noncoding RNAs from HHV-6B in this region (22). In 2014, a group from Israel concluded that the finding of processed pri-miRNA in supraspliceosomes brought further support to the cross talk between the splicing and miRNA (19–21) processing machinery (23). Pri-miRNAs are the precursors of pre-miRNAs, which are themselves the precursors of miRNAs. Pri-miRNAs are hairpin structures of about 70 nucleotides, with a 5′-cap and a 3′-poly(A) tail at the respective extremities. Therefore, the large number of sequencing reads and the numerous splicing events that we observed in the vicinity of the OriLyt region likely represent pri-miRNAs, which can be processed to produce many miRNAs. A long noncoding RNA (lncRNA1) found in this region was previously identified as the most highly expressed RNA in HHV-6B (15).

From the RNA-seq analysis at each of the infection time points, we obtained a heat map of the relative expression of each ORF described in the annotated HHV-6B genome (accession number AF157706) (Fig. 2). From the 9-h time point in the presence of CHX and the 72-h time point in the presence of PAA, we could match the majority of ORFs described in the annotated genome to their previously defined kinetic class, immediate-early (IE), early (E), and late (L) genes (Fig. 2 and Table 3). We then compared the kinetic classes obtained with those from two previous studies conducted on HHV-6B (Table 3) (24, 25). Of the 97 ORFs described in the annotated genome, 50 ORFs were newly classified or classified as previously mentioned by other studies (Table 3). Of the 47 remaining ORFs, 22 were classified as E genes, although these ORFs were previously classified as L genes in other studies (Table 3, green ORF). In general, the expression of E genes is not dependent on viral DNA replication (26). However, the accumulation of some E genes is enhanced by viral DNA replication; these genes are called early late genes. On the other hand, the expression of leaky late genes is delayed compared with that of E genes, and only true late genes can be identified by their “nonexpression” in the presence of PAA. The high sensitivity of RNA-seq used to analyze our data makes it difficult to distinguish early late genes from leaky late genes. Thus, our preference was to classify ORF transcripts that were detected 72 h postinfection in the presence of PAA as E genes. Nine ORFs from the remaining 25 were assigned to the L kinetic class, while other studies have classified these same genes as E genes (Table 3, orange ORF). Our results suggest these genes should be classified as L genes, as no transcription was observed for these genes at 72 h postinfection in the presence of PAA. Moreover, it was demonstrated that the hCMV homologs of HHV-6B U71 and U82 belong to the L gene kinetic class (Table 3) (27, 28). Four ORFs of the remaining 16 correspond to the B1, B5, B6, and B9 genes found only in the HHV-6B genome (Table 3, dark blue ORF). Conflicting reports exist regarding the kinetic class of these B genes. We have classified 3 genes as L genes, as they were not detected in the presence of PAA at 72 h postinfection (B1, B5, and B9). The fourth gene, B6, was classified as an E gene, since this transcript was found in the presence of PAA but was not detected in the presence of CHX. Previous studies classified B6 as an IE or a biphasic (IE and L) gene; however, these studies were conducted in different cell lines or with different HHV-6B strains (24, 25). Four genes out of the remaining 12 were assigned to the IE kinetic class, since they were expressed at high levels in the presence of CHX at 6 h postinfection (Table 3, gray ORF). These genes were previously assigned to the E or L kinetic class (24, 25). Two of the 8 remaining genes were previously classified as IE genes in other studies (Table 3, pink ORF). However, our study did not reveal any reads covering these genes in the presence of CHX (Fig. 2). Therefore, these genes were classified as members of the E kinetic class of genes. The remaining 6 genes were assigned to the IE kinetic class because some reads were detected in the presence of CHX and PAA (Fig. 2) (Table 3, blue ORF). These observations suggest that these genes can be classified as both IE and E genes. However, it is also possible that RNA from these genes is packaged within the virion and does not represent nascent transcription (Table 3, blue ORF). This is of particular relevance to the U2 gene, which showed a marked abundance of transcripts at 6 h postinfection (Fig. 2). A comparison of gene kinetic classes between hCMV and HHV-6B gene homologs is also presented in Table 3. We observed that most HHV-6B gene kinetic classes determined in this study are the same as those of their hCMV homologs.

**TABLE 3:**
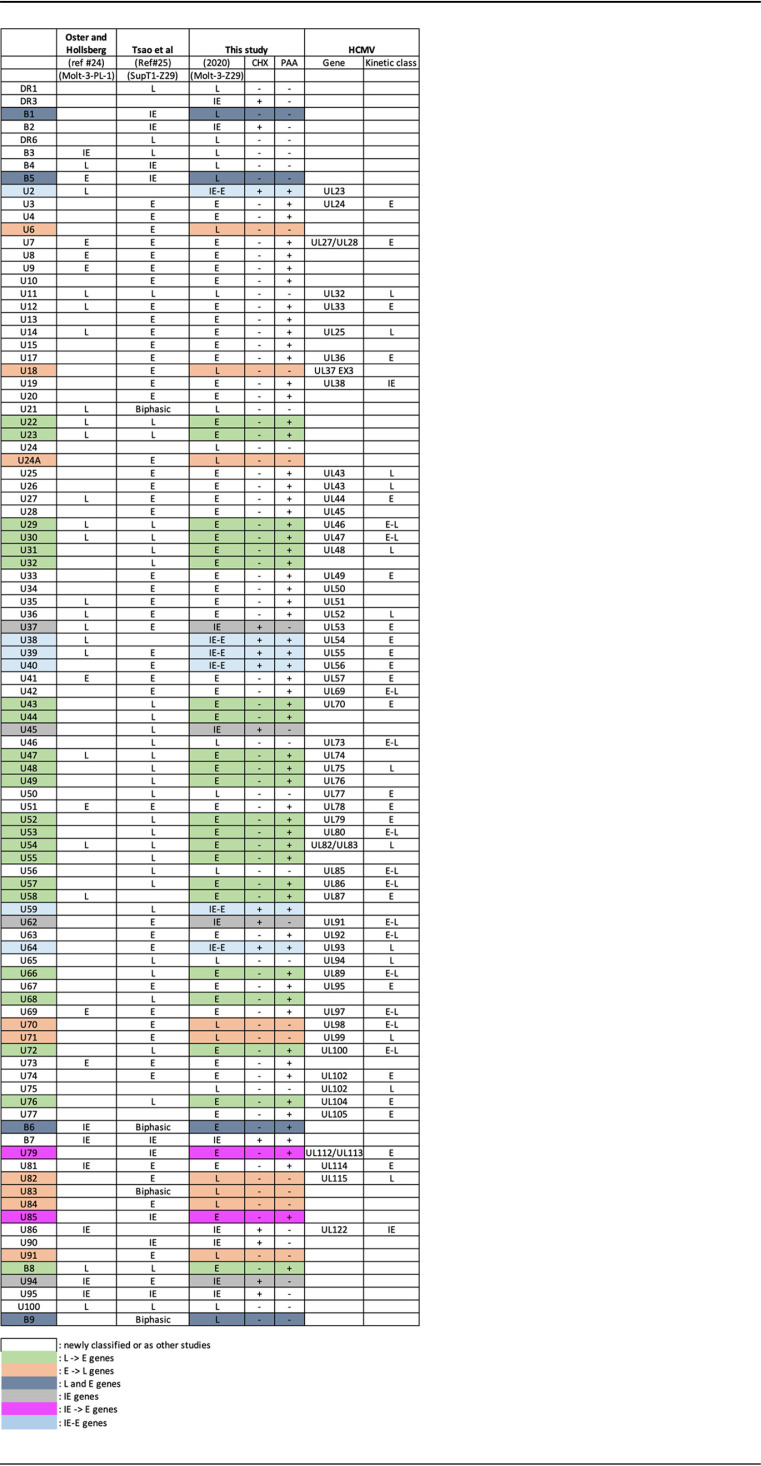
Comparison of the assigned kinetic classes obtained in this study with other studies and HCMV

**FIG 10:**
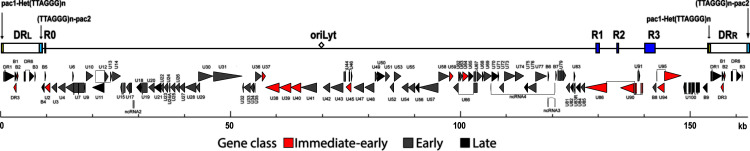
HHV-6B genomic organization. The upper section shows the positions of the major repeat elements (DR, R1, R2, and R3), the unique region, and the origin of replication. The ORFs are indicated in the lower section, and their colors reflect their kinetic classes. This figure was modified from the original version initially published by Dominguez et al. (11).

Next-generation sequencing combined with RNA-seq data bring us to deposit an updated version of the HHV-6B Z29 annotated genome. Our HHV-6B Z29 sequence was deposited under GenBank accession number MW536483. In this GenBank record and as shown in Table 4, we modified the annotated genome of HHV-6B to include mutations and new splicing regions described in this study. We also confirmed and included new spliced regions found in this study that were also found in previous studies: U19, U79, U83, U91 (18, 29), and U7-U8 and U12-U13, described in Finkel et al. (15). We identified a mutation that introduces a stop codon in U21 (Table 1). The U21 protein is truncated by 125 amino acids compared to the original U21 protein described in AF157706. This mutation was observed in our NGS data on HHV-6B DNA and in all RNA-seq data from the different time points mentioned in this study. However, it is not found in any other HHV-6B sequences in the database. As mentioned in a recent study on HSV-1 (30), we purposely did not rename any ORF in order to avoid causing confusion with previous work. Considerable work has been published on HHV-6B over the last 30 years, and this work forms the basis of our current knowledge. Thus, changing the name of an annotated ORF would cause unnecessary confusion in the field. Finkel et al. have chosen to rename the HHV-6B ORF and include their newly found internal ORF (iORF) and upstream ORF (uORF) in the annotation (15). Inclusion of the iORF and uORF in our modified annotated version of the AF157706 genome would be more suitable for comparison with historical nomenclature. Figure 10 summarizes the genomic organization of the HHV-6B Z29 genome.

**TABLE 4:** Comparison between AF157706 and MW536483 considering the results obtained in this study and previous studies

ORF	Strand orientation	AF157706		MW536483		Comment
		Start	Stop	Start	Stop	
DR1-L	+	583	841	583	841
955	2975	955	2975
DR3-L	−	2723	3325	2723	3325
B1-L	+	3022	3501	3022	3501
B2-L	+	3536	3775	3536	3775
DR6-L	+	5027	5330	5027	5330
6329	7203	6330	7204
B3-L	+	7349	7528	7350	7529
B4	−	8911	9492	8912	9493
B5	+	9522	9761	9523	9762
U2	−	9624	10715	9625	10716
U3	−	11045	12205	11046	12206
U4	−	12433	14040	12434	14041
U7	−	14159	15800	14160	15801
15890	16959	15891	16866
16972	18199
U6	+	15603	15809	15604	15810
U8	−	16963	18198	Part of U7
U9	−	18179	18493	18180	18494
U10	+	18543	20054	18544	20055
U11	−	19958	22534	19959	22535
U12	+	< 20801	20851	Noncoding exon
22636	22668	22618	22669	Partial noncoding exon; start at 22637
22746	23330	22747	23331
U13	+	<20801	20851	Noncoding exon
22618	22669	Partial noncoding exon; start at 22637
23856	24179	23854	24180
U14	+	24277	26109	24278	26110
U15	−	26143	26328	26144	26329
26500	26572	26501	26573
26732	27048	26733	27049
U17	−	27330	28105	27331	28106
28193	28421	28194	28422
U18	−	29601	30485	29602	30486
U19	−	30499	30541	Greninger et al. (18) and this study
30751	31920	30756	31921
U20	−	32145	33449	32146	33450
U21	−	33452	34954	33828	34955
U22	−	34851	35459	34852	35460
U23	−	35487	36386	35488	36387
U24	−	36511	36777	36512	36778
U24A	−	36796	36969	36797	36970
U25	−	36986	37936	36987	37937
U26	−	38044	38931	38045	38932
U27	−	38919	40019	38920	40020
U28	−	40135	42549	40136	42550
U29	−	42572	43471	42573	43472
U30	+	42999	46247	43000	46248
U31	+	46265	52498	46266	52499
U32	−	52572	52841	52573	52842
U33	−	52843	54255	52844	54256
U34	−	54206	55036	54207	55037
U35	−	55053	55373	55054	55374
U36	+	55372	56826	55373	56827
U37	+	56830	57624	56831	57625
U38	−	57670	60708	57671	60709
U39	−	60708	63200	60709	63201
U40	−	63154	65334	63155	65335
U41	−	65342	68740	65343	68741
U42	−	70103	71653	70104	71654
U43	−	71878	74460	71879	74461
U44	+	74501	75196	74502	75129
76012	76046
U45	−	75143	76273	75144	76274
U46	+	76047	76071	Noncoding exon
76346	76600	76283	76601	Partial noncoding exon; start at 76347
U47	−	76783	78999	76784	79000
U48	−	79265	81349	79266	81350
U49	+	81508	82266	81509	82267
U50	+	82043	83710	82044	83711
U51	+	83808	84713	83809	84714
U52	−	84732	85508	84733	85509
U53	+	85515	87101	85516	87102
U54	−	87336	88715	87337	88716
U55	−	88793	90271	88794	90272
U56	−	90272	91162	90273	91163
U57	−	91164	95201	91165	95202
U58	+	95213	97531	95214	97532
U59	+	97528	98580	97529	98581
U66	−	98577	99704	98578	99705
102912	103784	102913	103785
U62	+	99716	99979	99717	99980
U63	+	99921	100577	99922	100578
U64	+	100555	101883	100556	101884
U65	+	101840	102847	101841	102848
U67	+	<103390	103499	Noncoding exon
103756	104817	103775	104818	Partial noncoding exon; start at 103784
U68	+	104817	105161	104818	105162
U69	+	105164	106855	105165	106856
U70	+	106863	108329	106864	108330
U71	+	108266	108511	108267	108512
U72	−	108593	109627	108594	109628
U73	+	109640	111982	109641	111983
U74	+	111951	113939	111952	113940
U75	−	113974	114723	113975	114724
U76	−	114632	116620	114633	116621
U77	+	116415	118889	116416	118890
B6	+	119139	119378	119140	119379
B7	+	120836	121063	120836	121063
U79-1	+	121328	121376	Noncoding exon
121486	122116	121481	122116	Partial noncoding exon; start at 121486; Greninger et al. (18), Hill et al. (29), and this study
122213	122412	122213	122412	Greninger et al. (18), Hill et al. (29), and this study
122495	123106	122495	123106	Greninger et al. (18), Hill et al. (29), and this
U79-2	+	121328	121376	Noncoding exon; Hill et al. (29) and this study
121481	122116	Partial noncoding exon; start at 121486; Greninger et al. (18), Hill et al. (29), and this study
122213	122412	Greninger et al. (18), Hill et al. (29), and this study
122767	122784	Greninger et al. (18), Hill et al. (29), and this study
U81	−	123150	123917	123150	123917
U82	−	123993	124745	123993	124745
U83	+	124821	125162	124821	125162
U83R	−	124849	124989	Greninger et al. (18) and this study
125067	125178	Greninger et al. (18) and this study
U84	−	125268	126296	125268	126296
U85	−	126324	127202	126324	127202
U86	−	127339	131901	127340	131952
138044	138255
138343	138475	Partial noncoding exon; start at 138437; Gravel et al. (31) and this study
139281	139419	Noncoding exon; Gravel et al. (31) and this study
139578	139635	Noncoding exon; Gravel et al. (31) and this study
U90	−	135003	137932	135004	137933
138043	138254	138044	138255
138342	138436	138343	138475	Partial noncoding exon; start at 138437; Gravel et al. (31) and this study
139281	139419	Noncoding exon; Gravel et al. (31) and this study
139578	139635	Noncoding exon; Gravel et al. (31) and this study
U91	+	138591	138816	138592	138817	Greninger et al. (18) and this study
138926	139170	138914	138966	Greninger et al. (18) and this study
B8	−	141969	142766	141970	142767
U94	−	143093	144565	143095	144567
U95	+	142954	143000	Noncoding exon
144640	148278	144642	148280
U100-gQ2	−	148665	148911	148667	148913
149041	149169	149043	149171
149332	149341	149334	149537	Partial noncoding exon; start at 149506
U100-gQ1	−	148661	148913
149848	150008	149043	150010
150159	150272	150161	150274
150369	150476	150371	150478
150587	150664	150590	150667
150786	151121	150789	151124
151526	151807	151529	151810
151940	152325	151943	152365	Partial noncoding exon; start at 152328
152602	152726	Noncoding exon
158529	158770	Noncoding exon
B9	−	152932	153252	152875	153258
DR1-R	+	153904	154162	153910	154168
154276	156296	154282	156302
DR3-R	−	156044	156646	156050	156652
B1-R	+	156343	156822	156349	156828
B2-R	+	156857	157096	156863	157102
DR6-R	+	158348	158651	158354	158657
159650	160524	159657	160531
B3-R	+	160670	160849	160677	160856
ncRNA2	+	29157	29172
29288	29368
29454	29492
ncRNA3	−	118890	119002
119096	119158
120658	120753
ncRNA4	−	106777	106820
120658	120704

Our work is not without limitations. Although we are confident that most of the viral transcripts were detected, some regions had limited, if any, coverage in our data set. These regions could be transcriptionally silent regions or express very low levels of transcripts below the limit of detection. Second, differences between our data and those of others could result from the use of different cell lines for infection. HHV-6B transcriptional regulation and patterns may differ slightly depending on the cell lines for infection. Indeed, this observation was made by Greninger et al. for the U79 spliced transcripts (18). Finally, differences in the HHV-6B viral strains used, or the number of times the virus was passaged (and in which cell type), may also have had an impact on the overall results.

In conclusion, our work provides an up-to-date analysis of the HHV-6B Z29 transcriptome that complements the work of Finkel et al. (15). The data generated should prove useful to generate a better understanding of the complex and highly regulated life cycle of herpesviruses.

## MATERIALS AND METHODS

### Cell lines and virus.

Molt-3 cells (CRL-1552; ATCC) were cultured in RPMI 1640 (Corning Cellgro, Manassas, VA, USA) supplemented with 10% fetal bovine serum (Corning Cellgro), HEPES, and Plasmocin at 5 μg/ml (InvivoGen, San Diego, CA, USA). HHV-6B (Z29 strain) was propagated in Molt-3 cells, as previously described (31).

### Infection.

Seventy million Molt-3 cells were infected with HHV-6B at a multiplicity of infection (MOI) of 0.5 for 4 h in 4 ml of supplemented RPMI 1640. Cells were then washed twice with 1× phosphate-buffered saline (PBS). DNA was extracted from 1,000,000 cells, and the remaining cells were resuspended at 500,000 cells/ml in supplemented RPMI 1640. Ten million cells were harvested at 6, 9, 12, 24, 48, and 72 h postinfection, and the cell pellets were stored at −80°C until extraction. DNA was extracted from 1,000,000 cells at 72 h postinfection. Ten million Molt-3 cells were incubated in the presence of 10 μg/ml cycloheximide (CHX) (MilliporeSigma Canada, Oakville, ON, Canada) for 30 min before HHV-6B infection at an MOI of 0.5. Cells were maintained in 600 μl for 4 h, washed twice with 1× PBS, and resuspended at 500,000 cells/ml in supplemented RPMI 1640 containing 10 μg/ml CHX. Cells were harvested at 9 h postinfection, and the pellet was stored at −80°C until extraction. Ten million Molt-3 cells were incubated in the presence of 100 μg/ml phosphonoacetic acid (PAA) (MilliporeSigma Canada) for 30 min before HHV-6B infection at an MOI of 0.5. Cells were maintained in 600 μl for 4 h and washed twice with 1× PBS. DNA was extracted from 1,000,000 cells, and the remaining cells were resuspended at 500,000 cells/ml in supplemented RPMI 1640 containing 100 μg/ml PAA. Cells were harvested at 72 h postinfection, and the pellet was kept stored at −80°C until extraction. DNA was extracted from 1,000,000 cells at 72 h postinfection in the presence of PAA. Ten million uninfected Molt-3 cells were also harvested as a negative control.

### DNA extraction and ddPCR.

DNA was extracted from cells using the QiaAMP DNA blood minikit as described by the manufacturer (Qiagen Inc.) and analyzed by ddPCR as previously described (32). In brief, 10 ng of gDNA from each time point was analyzed using primers and probes designed to detect the HHV-6B U67-U68 gene and the RPP30 reference cellular gene. Data were normalized to the corresponding genome copies of the cellular RPP30 gene and expressed as copies per cell (33).

Linear HHV-6B viral DNA was isolated as described above from cell-free viral stocks prepared as previously described (31). The HHV-6B DNA was used for next-generation sequencing using the Novaseq 6000 from Illumina (San Diego, CA, USA).

### RNA extraction.

Total RNA was isolated using the standard Qiazol protocol (Qiagen Inc., Toronto, ON, Canada). rRNA was removed using a Ribo-Zero gold rRNA removal kit (human/mouse/rat) by following the manufacturer’s instructions (Illumina Inc.).

### RNA-seq.

Libraries were generated for all conditions using the Ion Total RNA-Seq kit v2 (Thermo Fisher Scientific, Ottawa, ON, Canada) by following a standard protocol. Libraries were then sequenced on an Ion Torrent S5 sequencer (Thermo Fisher Scientific).

Sequencing returned 94 million reads with greater than 9.4 million reads per condition. Reads were aligned to the Homo sapiens reference genome GRCh38 and to the Z29 strain of HHV-6B (NCBI accession number AF157706). Alignments were performed using Bowtie2 v2.3.4.1, and junction prediction was determined using TopHat v2.1.1. Transcriptional and gene read counts were obtained with CLC Genomics Workbench v9.5.2 (Qiagen) using the RNA-seq analysis tool and the genome annotations mentioned above.

### Immunofluorescence.

Immunofluorescence was performed as previously described (34). In brief, HHV-6B-infected Molt-3 cells were deposited on a 10-well microscope slide, dried, and fixed in acetone at −20°C for 10 min. The following primary antibodies were used: rabbit-α-IE1-Alexa-488, mouse-α-P41 (NIH AIDS Reagent Program, Germantown, MD, USA), and mouse-α-DR6/7 (NIH AIDS Reagent Program). Goat α-mouse-Alexa-488 was used as the secondary antibody (Life Technologies Inc., Burlington, ON, Canada). Slides were observed at 63× using a spinning disc confocal microscope (Leica DMI6000B) and analyzed with the software Volocity 5.4.

### RT-PCR.

Ten million Molt-3 cells continuously infected with HHV-6B were harvested. Total RNA was isolated using the standard Qiazol protocol (Qiagen Inc.) and processed for mRNA analysis by reverse transcriptase PCR (RT-PCR). In brief, 1 μg of total RNA was reverse transcribed in a total volume of 20 μl using the Moloney murine leukemia virus reverse transcriptase. One-tenth of the cDNA was used for the amplification of specific splicing junctions, using the primers specified in Table 5. PCR products were amplified, using Q5 high-fidelity DNA polymerase (New England BioLabs Ltd., Whitby, ON, Canada), at 98°C for 1 min, 40 cycles of 10 s at 98°C, 30 s at 60°C, and 3 min at 72°C, and a final elongation step at 72°C for 2 min. PCR products were then run on a 2% agarose gel and visualized using a Gel Doc XR+ System (Bio-Rad Laboratories Canada Inc., Mississauga, ON, Canada).

**TABLE 5:** Oligonucleotides used in this study

Name	Primer sequence	Figure
U7 Ex2 reverse	5′-GGCCAGGCGATACAACAT-3′	5C
U7 Ex1 forward	5′-TTTGCGGACTGCGGTTAT-3′	5C
U12-U13 Ex2 forward	5′-GACACTGTCATTGAGCTGTCC-3′	6C–E
U12 Ex3 reverse	5′-CTCTCAATGATTCGTGCTGTCT-3′	6C
U13 Ex3 reverse	5′-AAATCACAGACAGGGACGATAA-3′	6E
U12-U13 Ex1 forward	5′-CGAGCAGTCACAGTTTCAGT-3′	6G
U12-U13 Ex2 reverse	5′-CTGGAGACTTACCAGGAGTTTG-3′	6G
U44 Ex1 forward	5′-TCTCCATGAGGAGACAGGATTA-3′	7C
U44 Ex2 reverse	5′-CCTCTTATCAACGCCCAATCT-3′	7C
U67 Ex1 forward	5′-GGATTCGAGAAACTCTGTGTATTG-3′	8C
U67 Ex2 reverse	5′-CCTTATCCTGCTCTGTGAGATT-3′	8C
U69R sncRNA forward	5′-TCTCTACAGTCGTCCGAGTATG-3′	9C
U69R sncRNA reverse	5′-TGCAGATTCATGCTTATGTAAACG-3′	9C
U67-U68 forward	5′-TTCCGGTATATGACCTTCGTAAGC-3′	3A
U67-U68 reverse	5′-GATGTCTCACCTCCAAATCTTTAGAAAT-3′	3A
U67-U68 probe	5′-FAM-ACATTATAT-ZEN-RTCGAAYYTGACRCTACCTTCCG-IABkFQ-3′	3A
hRPP30 forward	5′-GATTTGGACCTGCGAGCG-3′	3A
hRPP30 reverse	5′-GCGGCTGTCTCCACAAGT-3′	3A
hRPP30 Probe	5′-HEX-TCTGACCTG-ZEN-AAGGCTCTGCGCG-IABkFQ-3′	3A

### Sequencing.

Sanger sequencing and next-generation sequencing were performed at the facility located at the CHU de Québec-Université Laval Research Center.

### Data availability.

The annotated HHV-6B sequence has been deposited under the GenBank accession number MW536483. Sequencing data files have been deposited under the BioProject accession number PRJNA680783.
